# A new tool to screen patients with severe obstructive sleep apnea in the primary care setting: a prospective multicenter study

**DOI:** 10.1186/s12890-022-01827-0

**Published:** 2022-01-15

**Authors:** Patricia Peñacoba, Maria Antònia Llauger, Ana María Fortuna, Xavier Flor, Gabriel Sampol, Anna Maria Pedro-Pijoan, Núria Grau, Carme Santiveri, Joan Juvanteny, José Ignacio Aoiz, Joan Bayó, Patricia Lloberes, Mercè Mayos, the PASHOS Working Group, Leandra Domínguez Olivera, Pepi Valverde Trillo, MªÁngeles Santos Santos, Mª del Mar Farga Martínez, Montserrat Reverté Simó, Núria Argemí Saburit, Casimira Medrano Medrano, Marineus Puig García

**Affiliations:** 1 Sleep Unit, Department of Respiratory Diseases, Hospital de La Santa Creu I Sant Pau, Universitat Autònoma de Barcelona, Barcelona, Spain; 2 Department of Medicine, Universitat Autònoma de Barcelona, Barcelona, Spain; 3 Equip d’Atenció Primària Encants, Centre d’Atenció Primària (CAP) Maragall, Institut Català de La Salut, Barcelona, Spain; 4 CAP Chafarinas, Institut Català de La Salut, Barcelona, Spain; 5 Sleep Unit, Service of Pneumology, Hospital Universitari Vall d’Hebron, Universitat Autònoma de Barcelona, Barcelona, Spain; 6 Centro de Investigación Biomédica en Red Enfermedades Respiratorias (CIBERES), Instituto de Investigación Carlos III, Madrid, Spain; 7 Àrea Bàsica de Salut Gaudí, Consorci Sanitari Integral, Barcelona, Spain; 8 Sleep Unit, Department of Respiratory Medicine, Parc de Salut Mar-IMIM, Hospital del Mar, Barcelona, Spain; 9 Service of Pneumology, Hospital Dos de Maig, Consorci Sanitari Integral, Barcelona, Spain; 10 CAP Trinitat Vella, Institut Català de La Salut, Barcelona, Spain; 11 CAP El Clot, Institut Català de La Salut, Barcelona, Spain

**Keywords:** Obstructive sleep apnea, Continuous positive airway pressure (CPAP), Primary healthcare, Sleep unit, Screening model, Home sleep apnea test

## Abstract

**Background:**

The coordination between different levels of care is essential for the management of obstructive sleep apnea (OSA). The objective of this multicenter project was to develop a screening model for OSA in the primary care setting.

**Methods:**

Anthropometric data, clinical history, and symptoms of OSA were recorded in randomly selected primary care patients, who also underwent a home sleep apnea test (HSAT). Respiratory polygraphy or polysomnography were performed at the sleep unit to establish definite indication for continuous positive airway pressure (CPAP). By means of cross-validation, a logistic regression model (CPAP yes/no) was designed, and with the clinical variables included in the model, a scoring system was established using the β coefficients (*PASHOS Test*). In a second stage, results of HSAT were added, and the final accuracy of the model was assessed.

**Results:**

194 patients completed the study. The clinical test included the body mass index, neck circumference and observed apneas during sleep (AUC 0.824, 95% CI 0.763–0.886, *P* < 0.001). In a second stage, the oxygen desaturation index (ODI) of 3% (ODI3% ≥ 15%) from the HSAT was added (AUC 0.911, 95% CI 0.863–0.960, *P* < 0.001), with a sensitivity of 85.5% (95% CI 74.7–92.1) and specificity of 67.8% (95% CI 55.1–78.3).

**Conclusions:**

The use of this model would prevent referral to the sleep unit for 55.1% of the patients. The two-stage PASHOS model is a useful and practical screening tool for OSA in primary care for detecting candidates for CPAP treatment.

*Clinical Trial Registration* Registry: ClinicalTrials.gov; Name: PASHOS Project: Advanced Platform for Sleep Apnea Syndrome Assessment; URL: https://clinicaltrials.gov/ct2/show/NCT02591979; Identifier: NCT02591979. Date of registration: October 30, 2015.

**Supplementary Information:**

The online version contains supplementary material available at 10.1186/s12890-022-01827-0.

## Background

Obstructive sleep apnea (OSA) is a breathing disorder with high and increasing prevalence [1] and important deleterious effect on the patient’s health and quality of life [2–5]. There is a wide consensus about the need for involvement of all healthcare levels especially primary care in the management of OSA [6, 7]. The participation of primary care professionals may contribute to improve underdiagnosis of OSA [8], which is particularly relevant because of conclusive evidence of the beneficial effects of continuous positive airway pressure (CPAP) on the overall health and prognosis of the patients [9]. In this context, primary care professionals play a double role. On one hand, to identify patients with severe OSA who should be referred to a specialized sleep unit for treatment and follow-up. On the other hand, to identify patients with mild-moderate OSA who can be managed in primary care, thus preventing unnecessary referrals and workload for sleep units. Different strategies with variable results have been proposed to meet this double objective, including the use of questionnaires alone [10–13] or combined with simplified models of home sleep apnea test (HSAT) [14–16], or strategies in which primary care physicians assume a central role in the care of patients with OSA [17–21]. The ideal model probably includes a coordinated network between different levels of care, adapted to the health care characteristics of the region and workload of the different settings, and able to be applicable to the broad spectrum of OSA phenotypes.

The PASHOS project (PASHOS is the Spanish acronym of Advanced Platform for Sleep Apnea Assessment) is a multicenter study conceived for the implementation of an inter-level working model, coordinated between primary care and sleep units. Previous development of the PASHOS project included specific training of primary care professionals (physicians and registered nurses), establishment of inter-level network tools [22] and analysis of primary care and sleep unit agreement in management decisions for OSA patients [23]. A moderate level of concordance on diagnostic classification between primary care physicians and sleep specialists was found, there was substantial agreement in patients with severe OSA who were candidates for CPAP therapy [23]. However, the overall sensitivity for detecting candidates for CPAP treatment by primary care physicians was only 62.2% [23]. Therefore, the development of a screening tool that would help in the clinical assessment and improve the ability to identify patients with OSA in the primary care setting seems justified. The objective of this study was to develop a two-stage screening model based on a clinical questionnaire and a HSAT, to detect patients with severe OSA who are candidates for treatment with CPAP.

## Methods

### Design and participants

This was a prospective, multicenter study with the participation of six primary health care centers and four sleep units from the urban area of Barcelona (Spain). The methodology of the study has been previously described in detail [22]. The PASHOS project has been approved by the Clinical Research Ethics Committee of the 10 participating centers, and written informed consent was obtained from all patients. The study was registered at ClinicalTrials.gov (identifier NCT02591979).

Between May 2015 and May 2018, men and women aged ≥ 18 and ≤ 75 years who visited a participating primary care center for any reason, were consecutively included according to a randomized schedule. Exclusion criteria were as follows: previous diagnosis of OSA, chronic insomnia (< 5 h of sleep/day), cognitive impairment or psychophysical inability to perform the HSAT, acute or unstable cardiovascular or cerebrovascular disease, neuromuscular disease, moderate-to-severe chronic obstructive pulmonary disease (COPD) (FEV_1_/FVC < 0.7% and FEV_1_ < 50% predicted), and relevant respiratory comorbidity that may interfere with arterial blood saturation measurements.

### Study procedures and data collection

The two-stage study design included a clinical assessment at the primary care center and a HSAT. At the patient’s visit in the primary care center, the following data were recorded: sociodemographic (age and sex), anthropometric (weight, height, body mass index [BMI], and neck, waist and hip circumference), history of relevant diseases and cardiovascular risk factors, forced spirometry, clinical history directed to assessment of sleep breathing disorders, daytime sleepiness using the Epworth sleepiness scale [24], the Berlin questionnaire [25], OSA50 questionnaire [14] and the STOP-Bang sleep apnea questionnaire [11].

The clinical probability of OSA based on scores of the Berlin questionnaire [25] was used to assess the prevalence of patients with low and high risk of OSA. To ensure a balanced sample between patients with low and high risk, according to the method proposed by Chai-Coetzer et al. [14] and considering an expected prevalence of OSA of 25–30% in primary care [25], all patients in the high-risk category and, randomly, 1 out of 2 patients in the low-risk underwent a HSAT. In all cases, sleep studies were performed using a self-applied Sibelmed Screen & Go® polygraph device (Bitmed, Sibelgroup, Barcelona, Spain), with a 2-channel monitor: nasal cannula for airflow measure and oximetry. The device also provided body position. The primary care nurses assessed the quality of the HSAT and removed the periods of poor signaling (artifacts or lost signal). The minimum valid recording time was 5 h, and the sleep study was repeated if a poor signal acquisition was detected or the recording time was less than 5 h. Variables recorded included peripheral oxygen saturation (SpO_2_), falls in SpO_2_ ≥ 3% (oxygen desaturation index—ODI3%) and ≥ 4% (ODI4%) per hour of recording, and time spent in the supine position. A hypopnea was defined as an airflow reduction of ≥ 30% but < 90% lasting at least 10 s, with a ≥ 3% drop in oximetry, and an apnea was defined as an absence of airflow or ≥ 90% reduction for ≥ 10 s. Cut-off points for AHI were 5–15 for mild OSA, > 15 and < 30 for moderate OSA, and ≥ 30 for severe OSA.

All patients independent of the clinical probability of OSA and within 3 months after HSAT, were referred to the sleep unit and underwent a respiratory polygraphy or conventional polysomnography according to standard practice [26, 27]. Specialists at the sleep unit with all clinical documentation available established a final diagnosis and therapeutic decision regarding whether or not patients were candidates for CPAP therapy. The diagnosis of OSA and indication of CPAP treatment were made according to clinical practice guidelines [26, 27].

### Statistical analysis

The sample size was estimated considering a minimum prevalence of OSA of 25% in the population attended to in the primary care setting [25]. Assuming a loss of 15% at follow-up and an alpha error of 5%, a total sample of 162 patients was needed to detect a sensitivity of 90% in the validation process. Categorical variables are expressed as frequencies and percentages, and continuous variables as mean, standard deviation (SD) and 95% confidence interval (CI). Bivariate analysis included the chi-square test (χ^2^) test or the Fisher’s exact probability test for categorical data, and the Student’s *t* test or the Mann–Whitney *U* test for continuous data according to the conditions of application.

Development of the screening predictive model was made according to a cross-validation procedure, with the whole sample available for estimation of the model and further validation. Thus, all clinical variables were included in a multivariate logistic regression analysis, in which indication of CPAP treatment (yes/no) made by sleep specialists was the dependent variable. Categorical variables of clinical questionnaires were dichotomously recodified. In order to simplify the predictive clinical screening model and facilitate its applicability in daily practice, a system of stratification and scoring according to β coefficients of the logistic regression model [13] was developed. The variance inflation factor (VIF) was used to assess multicollinearity of the model, with VIF < 5 indicating a complete absence of collinearity [28]. Diagnostic accuracy of clinical variables and HSAT was assessed with the area under the curve (AUC) of the receiver operating characteristic (ROC) curve.

A first analysis was performed including only the clinical variables. For each of the final scores, sensitivity, specificity, positive and negative predictive values, overall accuracy, likelihood ratio, odds ratio, and the post-test probability with the 95% CI were calculated. The cut-off point of maximum sensitivity and moderate specificity was chosen. The same data was calculated for the results of the Epworth sleepiness scale, the Berlin questionnaire, the STOP-Bang sleep apnea questionnaire, and the OSA50 questionnaire. In a second analysis, the ROC curve and the AUC were recalculated after including variables collected in the HSAT, such as apnea–hypopnea index (AHI), ODI3% and ODI4%. Statistical significance was set at *P* < 0.05. Statistical analyses were performed using the statistical software IBM-SPSS version 26.0 for Windows.

## Results

### Characteristics of participants

**Fig. 1:**
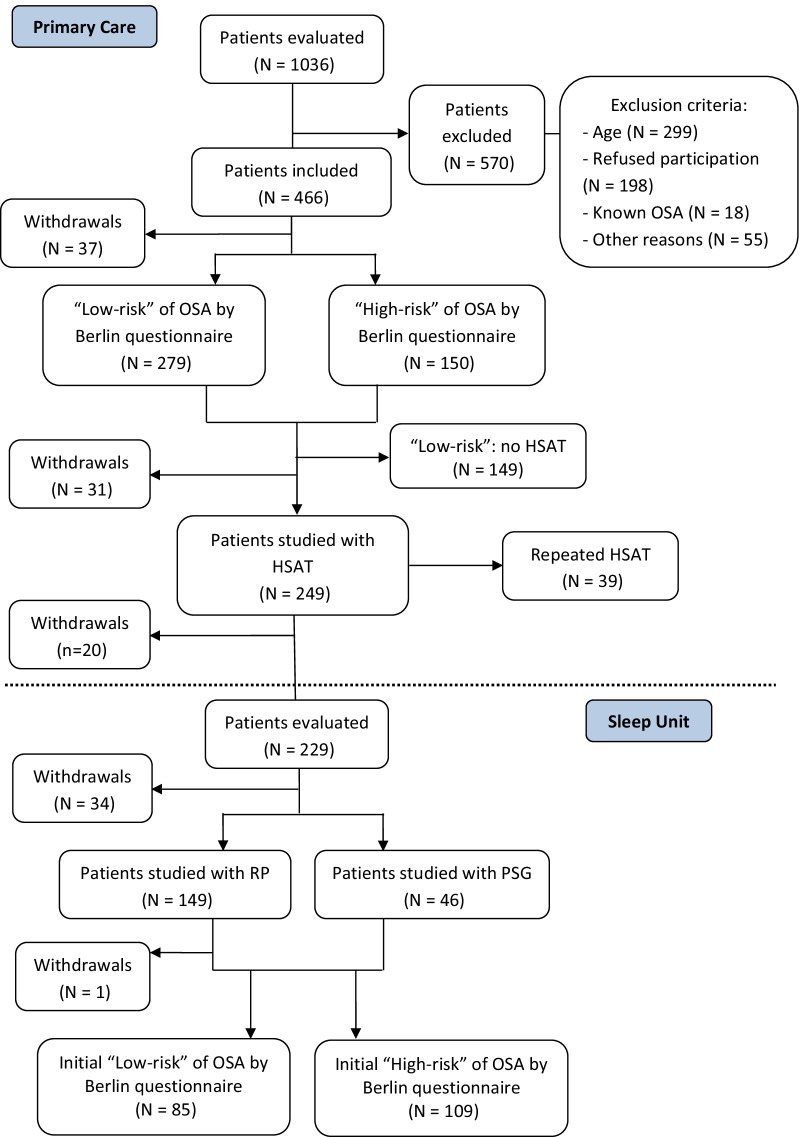
Flow chart of the study population (OSA: obstructive sleep apnea; HSAT: home sleep apnea test; RP: respiratory polygraphy; PSG; polysomnography)

A total of 1036 patients were assessed by primary care physicians, 466 (45%) of which were eligible and agreed to take part in the study. A total of 249 (53.4%) patients underwent HSAT. There were no significant differences between patients with low clinical probability of OSA based on scoring of the Berlin questionnaire that were randomized to or not to perform a HSAT (Table S1, Supplementary material). Finally, 194 (41.6%) patients completed the second stage with a respiratory polygraphy or conventional polysomnography at the sleep unit, and the definitive diagnosis and therapeutic decision was established. Of the total 194 patients who completed the study, 126 (64.9%) were not considered candidates for CPAP therapy, whereas the remaining 68 (35.0%) were candidates for CPAP treatment. The flow chart of the study population is shown in Fig. 1. The clinical characteristics of patients in the overall study population and in the groups of candidates and non-candidates for CPAP therapy are shown in Table 1.

**Table 1:** Characteristics of patients and results of sleep studies according indication of CPAP treatment by the sleep specialist

Variables	All patients (n = 194)	Non-candidates for CPAP (n = 126)	Candidates for CPAP (n = 68)	*P *value
Men:women	106:88	56:70	50:18	< 0.001
Age, years, mean (SD)	55.9 (12)	55.1 (12.3)	57.4 (11.4)	0.202
Anthropometric variables, mean (SD)				
Body mass index, kg/m^2^	29.2 (5.1)	27.7 (4.5)	32 (5.2)	< 0.001
Neck circumference, cm	38.2 (4.6)	36.7 (4.2)	41.1 (4)	< 0.001
Waist circumference, cm	98.7 (13.7)	94 (12.6)	107.4 (11.2)	< 0.001
Hip circumference, cm	106.6 (10.5)	103.8 (10)	111.8 (9.4)	< 0.001
Waist-hip ratio	0.92 (0.11)	0.9 (0.1)	0.94 (0.12)	0.008
Comorbidities				
Hypertension	68 (35.1)	36 (28.6)	32 (47.1)	0.010
Diabetes mellitus	35 (18)	23 (18.3)	12 (17.6)	0.008
Dyslipidemia	70 (36.1)	44 (34.9)	26 (38.2)	0.656
Depression	18 (9.3)	10 (7.9)	8 (11.8)	0.381
Anxiety	35 (18)	23 (18.3)	12 (17.6)	0.916
Stroke	2 (1)	2 (1.6)	0 (0)	0.542
Arrhythmia	7 (3.6)	2 (1.6)	5 (7.4)	0.053
Peripheral artery disease	8 (4.1)	4 (3.2)	4 (5.9)	0.365
Hypothyroidism	12 (6.2)	7 (5.6)	5 (7.4)	0.620
Heart disease	13 (6.7)	6 (4.8)	7 (10.3)	0.141
Active smoking	44 (22.7)	27 (21.4)	17 (25)	0.847
Alcohol consumption	69 (35.9)	38 (30.4)	31 (46.3)	0.029
Spirometry, mean (SD)				
FEV_1,_ %	90.6 (13.8)	91.5 (14)	89 (13.5)	0.236
FVC, %	92 (14.9)	92.3 (15.1)	91.5 (14.6)	0.730
Epworth sleepiness scale, mean (SD)	7.8 (5)	7.2 (4.6)	8.8 (5.5)	0.031
Patients with low/high risk of OSA				
Berlin questionnaire, n = 194	85/109	65/61	20/48	
STOP-Bang, n = 193	57/136	49/76	8/60	
OSA50, n = 185	44/141	39/80	5/61	
Epworth scale score ≥ 11, n = 193	142/51	101/24	41/27	
HSAT, mean (SD)				
Recording time, min	414 (84.8)	418.3 (87.4)	406 (79.9)	0.334
AHI	13.7 (15.4)	7.7 (7.5)	24.9 (19.5)	< 0.001
AHI supine	24.2 (23.2)	15 (16)	41.3 (24.8)	< 0.001
Oxygen saturation (SpO_2_)	92.5 (3.2)	93.2 (2.2)	91.3 (4.1)	< 0.001
CT90%	11.3 (19.5)	7.6 (18)	18.3 (20.6)	< 0.001
ODI3%	18.5 (16.3)	10.9 (8.3)	32.7 (17.9)	< 0.001
ODI4%	12.9 (14.7)	6.1 (6.1)	25.8 (17.4)	< 0.001
RP or conventional PSG, mean (SD)				
Recording time, min	463 (58.2)	462.3 (50.8)	464.2 (70.2)	0.829
AHI	21.3 (19.7)	10.1 (7.4)	42.1 (18.5)	< 0.001
AHI supine	24.3 (21.6)	12.9 (10.6)	47.2 (20)	< 0.001
Oxygen saturation (SpO_2_)	93.5 (2)	94.1 (1.7)	92.2 (2)	< 0.001
CT90%	7.1 (13.8)	2.9 (9.9)	16.4 (0.1)	< 0.001
ODI3%	18.9 (18.2)	8.6 (6.5)	34.9 (19.1)	< 0.001
ODI4%	13.3 (15.7)	5.2 (5.4)	27.6 (17.7)	< 0.001

Data expressed as frequencies and percentages in parenthesis unless otherwise stated. CPAP: continuous positive airway pressure; HSAT: Home sleep apnea test; SD: standard deviation; FEV_1_: forced expiratory value in one second; FVC: forced vital capacity; AHI: apnea–hypopnea index; CT90%: cumulative percentages of time at saturations below 90%; ODI3%: oxygen desaturation index of 3%; ODI4%: oxygen desaturation index of 4%; RP: respiratory polygraphy; PSG: polysomnography

### Model based on clinical variables (*PASHOS test*)

After logistic regression analysis (candidate for CPAP treatment, yes or no) and taking into account all clinical data, the final model included the following three variables: BMI, neck circumference, and the question “Have you been told that you repeatedly stop breathing when asleep?”. The collinearity between these three variables was excluded. The AUC of the ROC curve was 0.824 (95% CI 0.763–0.886). Results of the logistic regression analysis and scores assigned to each variable are shown in Table 2. The final screening questionnaire, named the *PASHOS Test* (Table S2, Supplementary material), was scored between 0 and 11 (higher scores indicated higher probability of CPAP treatment for OSA). The AUC of the ROC curve for discriminating patients who were candidates or not for CPAP treatment was 0.824 (95% CI 0.764–0.884), almost identical to that of the regression model. The diagnostic accuracy of the test according to different cut-off values is shown in Table 3. A cut-off value of ≥ 5 classified a patient as candidate for CPAP treatment with a sensitivity of 92.6% (95% CI 83.9–96.8) and a specificity of 53.2% (95% CI 44.5–61.7), so that with a test score < 5, only 5 patients were excluded who were finally candidates for CPAP treatment.

**Table 2:** Results of multivariate analysis with stratification of variables and scores assigned to each variable in the *PASHOS Test*

Variables	Coefficient (β)	Standard error	Wald (χ^2^)	*P *value	Odds ratio (95% CI)	Scores (*PASHOS Test*)
Neck circumference, cm						
≤ 35	Reference					0
> 35 and ≤ 41	1.472	0.545	7.300	0.007	4.359 (1.5–12.68)	3
> 41	2.466	0.605	16.621	< 0.001	11.771 (3.6–38.47)	5
BMI, kg/m^2^						
≤ 26	Reference					0
> 26 and ≤ 30	1.215	0.585	4.316	0.038	3.369 (1.07–10.6)	2
> 30 and ≤ 33	1.587	0.610	6.763	0.009	4.887 (1.48–16.15)	3
> 33	1.949	0.632	9.498	0.002	7.021 (2.03–24.24)	4
Breathing pauses during sleep						
No	Reference					0
Yes	0.942	0.379	6.171	< 0.001	2.566 (1.22–5.4)	2

BMI: body mass index; CI: confidence interval

**Table 3:** Diagnostic accuracy of the *PASHOS Test* according to different cut-off values

*PASHOS Test* cut-off value	Sensitivity % (95% CI)	Specificity % (95% CI)	Positive predictive value % (95% CI)	Negative predictive value % (95% CI)	Overall accuracy % (95% CI)	Positive likelihood ratio (95% CI)	Negative likelihood ratio (95% CI)	Odds ratio (95% CI)	Post-test probability % (95% CI)
≥ 2	98.5 (92.1–99.7)	11.9 (7.3–18.7)	37.6 (30.9–44.9)	93.8 (71.1–98.9)	42.2 (35.5–49.3)	1.11 (1.04–1.2)	0.12 (0.02–0.92)	9.05 (1.17–70.1)	31.2 (35.5–38.8)
≥ 3	98.5 (91.1–99.7)	27.0 (20–35.3)	42.1 (34.7–49.9)	97.1 (85.5–99.5)	52.1 (45.1–59.0)	1.35 (1.21–1.51)	0.05 (0.01–0.39)	24.76 (3.3–185.4)	41.7 (39.0–44.4)
≥ 4	95.6 (87.8–98.5)	46.0 (37.6–54.7)	48.9 (40.5–57.3)	95.1 (86.5–98.3)	63.4 (56.4–69.9)	1.77 (1.5–2.1)	0.1 (0.03–0.29)	18.48 (5.8–58.2)	48.4 (44.2–52.6)
≥ 5	92.6 (83.9–96.8)	53.2 (44.5–61.7)	51.6 (42.9–60.3)	93.1 (84.8–97.0)	67.0 (60.1–73.2)	1.98 (1.62–2.41)	0.14 (0.06–0.33)	13.31 (5.54–37)	51.1 (46.2–56.1)
≥ 6	79.4 (68.4–87.3)	69.8 (61.3–77.2)	58.7 (48.5–68.2)	86.3 (78.3–91.6)	73.2 (66.6–78.9)	2.63 (1.97–3.53)	0.29 (0.18–0.48)	8.93 (4.46–17.9)	58.2 (51.0–65.1)
≥ 7	70.6 (58.9–80.1)	77.0 (68.9–83.5)	62.3 (51.2–73.3)	82.9 (75.1–88.7)	74.7 (68.2–80.3)	3.07 (2.15–4.37)	0.38 (0.26–0.56)	8.02 (4.14–15.6)	61.9 (53.2–69.8)
≥ 8	52.9 (41.2–64.3)	85.7 (78.5–90.8)	66.7 (53.4–77.8)	77.1 (69.5–83.3)	74.2 (67.6–79.9)	3.71 (2.29–6.01)	0.55 (0.42–0.71)	6.75 (3.4–13.4)	66.2 (54.7–76.1)
≥ 9	44.1 (32.9–55.9)	91.3 (85.0–95.1)	73.2 (58.1–84.3)	75.2 (67.8–81.3)	74.7 (68.2–80.3)	5.05 (2.71–9.44)	0.61 (0.49–0.76)	8.25 (3.81–17.8)	72.8 (58.9–83.3)
≥ 10	20.6 (12.7–31.6)	98.4 (94.4–99.6)	87.5 (64.0–96.5)	69.7 (62.6–75.9)	71.1 (66.4–77.1)	13.0 (3.04–55.4)	0.80 (0.71–0.91)	16.1 (3.5–73.2)	87.3 (61.6–96.7)
11	14.7 (8.2–25.0)	99.2 (95.6–99,9)	90.9 (62.3–98.4)	68.3 (61.2–74.6)	69.6 (62.8–75.6)	18.5 (2.4–141.7)	0.86 (0.78–0.95)	21.6 (2.7–172.4)	90.7 (56.2–98.7)

CI: confidence interval

**Fig. 2:**
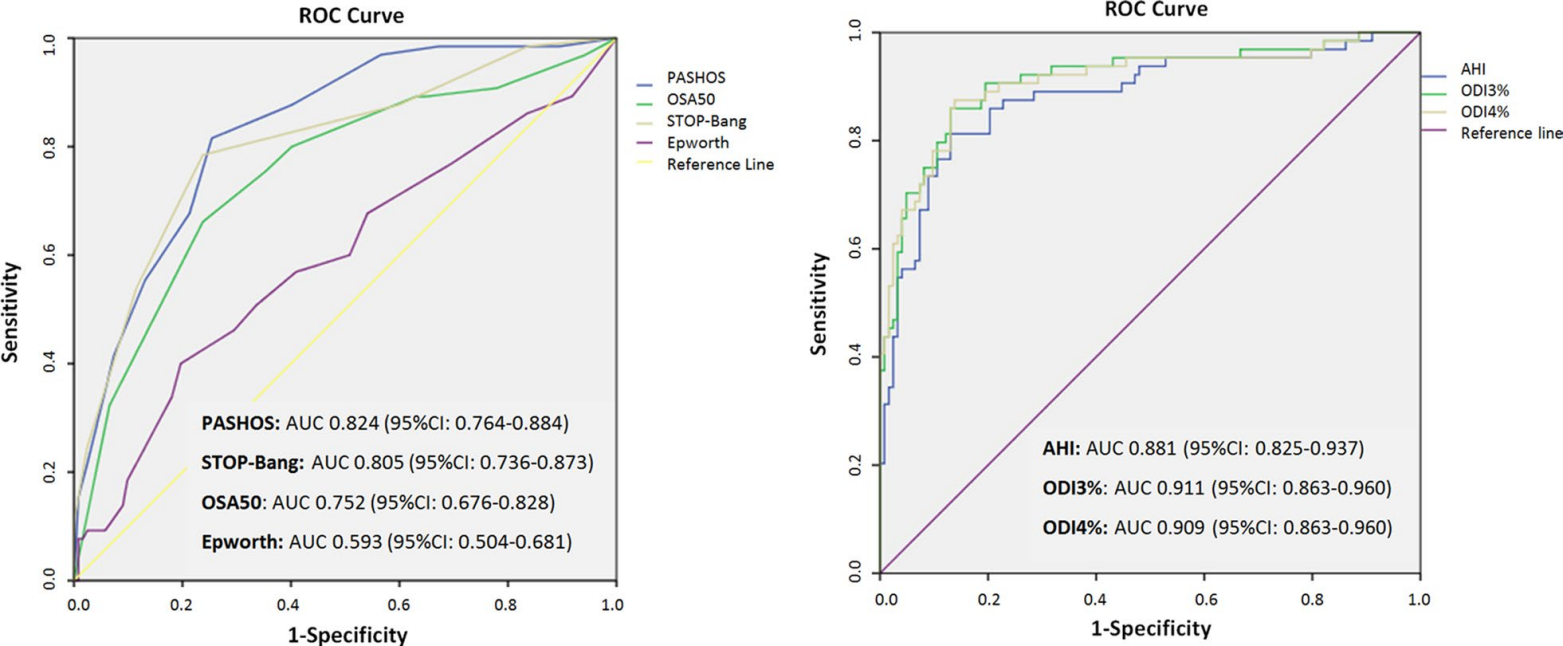
Diagnostic accuracy of the *PASHOS Test* for a cut-off value of ≥ 5 and different study questionnaires (left panel) as well as variables obtained with the home sleep apnea test (right panel) (AUC: area under the ROC curve; CI: confidence interval; AHI: apnea–hypopnea index; ODI3%: oxygen desaturation index of 3%; ODI4%: oxygen desaturation index of 4%)

The diagnostic accuracy of the *PASHOS Test* as compared with results of the Epworth sleepiness scale, Berlin questionnaire, STOP-Bang sleep apnea test, and OSA50 questionnaire is shown in Fig. 2. The AUC of the ROC curve was more favorable for the *PASHOS Test*, although differences were only statistically significant with the Epworth sleepiness scale. The sensitivity, specificity, positive and negative predictive values, and overall diagnostic accuracy for the *PASHOS Test* as compared to the study questionnaires are shown in Table S3 (Supplementary material). In this comparisons, the *PASHOS Test* showed the highest sensitivity (92.6%, 95% CI 83.9–96.8) and negative predictive value (93.1%, 95% CI 84.8–97.0).

### Model based on home sleep apnea test variables

The diagnostic accuracy of variables obtained in the HSAT were assessed and included in the logistic regression model. As shown in Fig. 2, the AUC of the ROC curve for ODI3% (0.911, 95% CI 0.863–0.960) was slightly higher than those corresponding to the AHI and ODI4%. The diagnostic accuracy of the *PASHOS* model in relation to cut-off values of the ODI3% between ≥ 10 and ≥ 30 is shown in Table S3 (Supplementary material). The sensitivity of the *PASHOS* model was 85.5% (95% CI 74.7–92.1) and the specificity 67.8% (95% CI 55.1–78.3) with an ODI3% of ≥ 15.

When the dependent variable in the logistic regression model was an AHI ≥ 30 obtained in the final respiratory polygraphy or conventional polysomnography, it was shown that the model included the same predictive variables and very similar values of the AUC of the ROC curve (0.819, 95% CI 0.753–0.885).

Based on the final decision taken by sleep specialists, the *PASHOS Test* with a cut-off value of ≥ 5 classified correctly 67 patients (34.5%) who were non-candidates for CPAP therapy, with a further 40 patients (20.6%) when an ODI3% of ≥ 15 was added (Table 4).

**Table 4:** Classification of patients according to results of the *PAHOS Test* alone and combined with ODI3%

Variable	Final indication of CPAP treatment by sleep specialists		Total patients
	No	Yes	
*PASHOS Test* ≥ 5			
CPAP treatment			
No	67 (34.5%)	5 (2.6%)	72
Yes	59 (30.4%)	63 (32.5%)	122
Total patients	126	68	194
*PASHOS model* (Test ≥ 5 and ODI3% ≥ 15)			
CPAP treatment			
No	40 (33.1%)	10 (7.4%)	50
Yes	19 (15.7%)	53 (43.8%)	72
Total patients	59	63	122

CPAP: continuous positive airway pressure; ODI3%: oxygen desaturation index of 3%

## Discussion

This study proposes a two-stage screening model for OSA developed in non-selected patients attended to in the primary care setting. This model includes a questionnaire (*PASHOS Test*) based on three clinical variables (BMI, neck circumference, breathing pauses during sleep) that in case of positivity (cut-off ≥ 5 points) is complemented by a HSAT. This model, easy to use in the clinical setting, has been able to identify patients at high risk of severe OSA who are candidates for CPAP treatment and should be referred to a sleep unit with a diagnostic accuracy higher than 80%. On the other hand, the use of the model would prevent referral to the sleep unit for 55.1% of patients with low to moderate risk of OSA. These patients could continue to be attended to in primary care, as a similar management model to other chronic diseases such as asthma or COPD.

Patients were recruited in the primary care setting independent of the reason for consultation and suspicion of OSA. In a second stage, the HSAT was performed in all patients in the OSA high-risk category of Berlin questionnaire and, randomly, 1 out of 2 patients in the low-risk category. However, results are unlikely to be affected by this randomization, since differences between patients randomized to or not to the sleep study were not found. Our approach is similar to the two-stage model developed by Chai-Coetzer et al. [14] based on 157 patients attending their primary care physician, for any reason, at six primary care clinics in rural and metropolitan regions of South Australia. The screening questionnaire in this study included snoring, waist circumference, witnessed apneas and age, and was followed by a HSAT. This two-stage diagnostic model showed a sensitivity of 97% and specificity of 87% [14]. In this study, as in other previous studies carried out in selected populations [15, 16], the predictive capacity of the model was higher, which may be explained by the fact that they included a HSAT in all patients, independently of the clinical variables. In our opinion, the indication of HSAT without any previous clinical filter may impose a work overload for primary care, transferring the problem of waiting lists to this setting and limiting its applicability. Other models of integrated management of patients with high clinical probability of OSA in primary care have been validated, with non-inferior mid-term results to conventional management in specialized sleep units [17–21]. These results, however, although they offer evidence that non-sleep specialists are capable of providing care to patients with OSA, have been questioned for difficulties of implementation in real-world clinical practice given the work overload in primary care [29].

The two-stage screening model proposed in the present study, includes a short, simple and easy to administer questionnaire (*PASHOS Test*) with a high sensitivity (93%) and negative predictive value of 93% that, in practice, correctly identified subjects at low risk of OSA in 34.5% of the study sample. The association of the *PASHOS Test* with ODI3% in a second stage increases the specificity and positive predictive value of the model, avoiding referral to sleep specialists to further 21% of patients. The *PASHOS Test* includes only three variables but shows sensitivity and negative predictive values similar or slightly higher than clinical questionnaires usually recommended for OSA screening, such as the Berlin questionnaire [10], OSA50 [14], and STOP-Bang [11], and clearly higher than the Epworth sleepiness scale, which shows a high specificity but a low sensitivity for detecting OSA [12].

The HSAT was performed using a 2-channel monitor with a nasal cannula for airflow measure and oximetry. In agreement with data reported in the study of Chai-Coetzer et al. [14], the ODI3% was the parameter with the highest diagnostic reliability. The primary care nurse assessed the quality of sleep studies. The studies were allowed to be repeated in case of poor signaling acquisition or even discarded when invalid recording.

The logistic regression model was calculated according to decision of treatment with CPAP (yes/no). However, this decision may include a subjective component so the AUC of the ROC curve was also calculated taking the AHI ≥ 30 as severe OSA obtained in the gold-standard sleep studies. The AUC obtained in this model was very similar, which supports the usefulness of the two-stage model for selecting candidates for CPAP treatment and patients with severe OSA according to AHI.

Strengths of the study include the multicenter design and the fact that the inclusion of patients was not limited to those with a pretest high clinical probability of OSA, so that the results obtained can be applicable to a large phenotype spectrum of patients, but considering the limitations of the study. The limitations include the eligibility criterion of an upper age range of 75 years, which excludes a substantial percentage of patients attended to in primary care. Patients with a previous clinical diagnosis of insomnia are also excluded, which may have contributed to the underdiagnosis of patients with OSA primarily with complaints of sleep disruption, and it is already known the existence of a clinical cluster of OSA related to a high prevalence of hypertension, diabetes, and cardiovascular disease [30]. Also, in contrast to other studies [14–19, 21], the definite diagnosis was not made using polysomnography in all patients [22]. Although respiratory polygraphy may underestimate the diagnosis of OSA, our model was adapted to routine clinical practice and recommendations of current clinical practice guidelines [27].

## Conclusion

This two-stage screening model that includes a short clinical questionnaire (*PASHOS Test*) and results obtained by a HSAT, is a useful approach in which primary care professionals have an important role in the management of OSA. With a previous adequate training program and in coordination with sleep specialists, unnecessary referrals of patients who are non-candidates for CPAP with mild-moderate OSA could be prevented.

## Supplementary Information

**Additional file 1: Table S1.** Characteristics of patients at low risk of OSA based on scores of the Berlin questionnaire randomized or not randomized to home sleep apnea test (HSAT). **Table S2.** *PASHOS Test.* **Table S3.** Diagnostic accuracy of the *PASHOS Test* as compared with the study questionnaires and with the addition of ODI3%.

## Data Availability

Study data are available from the corresponding author upon request.

## Abbreviations

AHI: Apnea–hypopnea index
AUC: Area under the curve
BMI: Body mass index
CI: Confidence interval
COPD: Chronic obstructive pulmonary disease
CPAP: Continuous positive airway pressure
CT90%: Percentage of time with oxygen saturation below 90%
FEV1: Forced expiratory volume in 1 s
FVC: Forced vital capacity
HSAT: Home sleep apnea test
ODI3%: Oxygen desaturation index of 3%
ODI4%: Oxygen desaturation index of 4%
OSA: Obstructive sleep apnea
PSG: Polysomnography
ROC: Receiver operating characteristic
RP: Respiratory polygraphy
SD: Standard deviation
SpO_2_: Peripheral oxygen saturation
VIF: Variance inflation factor
