# Supplementary material for: A new tool to screen patients with severe obstructive sleep apnea in the primary care setting: a prospective multicenter study
Source: BMC Pulm Med. 2022 Jan 15;22:38. doi: 10.1186/s12890-022-01827-0 (PMC8761286; doi:10.1186/s12890-022-01827-0)
Supplement: Supplementary file 1 — Additional file 1: Table S1. Characteristics of patients at low risk of OSA based on scores of the Berlin questionnaire randomized or not randomized to home sleep apnea test (HSAT). Table S2. PASHOS Test. Table S3. Diagnostic accuracy of the PASHOS Test as compared with the study questionnaires and with the addition of ODI3%. [file 12890_2022_1827_MOESM1_ESM.docx]

**Supplementary material**

**Table S1.** Characteristics of patients at low risk of OSA based on scores of the Berlin questionnaire randomized or not randomized to home sleep apnea test (HSAT).

| Variables | All patients  (n = 279) | Randomized to  HSAT  (n = 130) | Not randomized to HSAT  (n = 149) | *P*  value |
| --- | --- | --- | --- | --- |
| Men:women | 135:144 | 66:64 | 69:80 | 0.457 |
| Age, years, mean (SD) | 55.9 (12) | 55.1 (12.3) | 57.4 (11.4) | 0.202 |
| Anthropometric variables, mean (SD) |  |  |  |  |
| Body mass index, kg/m^2^ | 54.3 (13.6) | 54.6 (13.6) | 54 (13.6) | 0.696 |
| Neck circumference, cm | 26.91 (4.5) | 26.71 (4.5) | 27.1 (4.5) | 0.486 |
| Waist circumference, cm | 36.5 (5.5) | 37 (4) | 36.2 (6.5) | 0.224 |
| Hip circumference, cm | 92.2 (13.3) | 92.9 (13.3) | 91.7 (13.3) | 0.447 |
| Waist-hip ratio | 102 (9.5) | 102.5 (9.5) | 101.5 (9.5) | 0.412 |
| Comorbidities |  |  |  |  |
| Hypertension | 72 (26.5) | 34 (27.2) | 38 (25.9) | 0.901 |
| Diabetes mellitus | 32 (11.5) | 19 (14.6) | 13 (8.7) | 0.123 |
| Dyslipidemia | 77 (27.6) | 40 (30.8) | 37 (24.8) | 0.268 |
| Depression | 22 (7.9) | 11 (8.5) | 11 (7.4) | 0.739 |
| Anxiety | 44 (15.8) | 15 (11.5) | 29 (19.5) | 0.070 |
| Stroke | 5 (1.8) | 2 (1.5) | 3 (2) | 1.000 |
| Arrhythmia | 11 (3.9) | 3 (2.3) | 8 (5.4) | 0.190 |
| Peripheral artery disease | 8 (2.9) | 5 (3.8) | 3 (2) | 0.479 |
| Hypothyroidism | 15 (5.4) | 5 (3.8) | 10 (6.7) | 0.290 |
| Heart disease | 11 (3.9) | 5 (3.8) | 6 (4) | 0.938 |
| Active smoking | 57 (20.4) | 29 (22.3) | 28 (18.8) | 0.748 |
| Alcohol consumption | 102 (37) | 50 (39.1) | 52 (35.1) | 0.500 |
| Spirometry, mean (SD) |  |  |  |  |
| FEV_1,_ % | 93.4 (14.6) | 93 (14.6) | 93.8 (14.6) | 0.622 |
| FVC, % | 93.8 (15.8) | 93.9 (15.3) | 93.7 (16.3) | 0.924 |
| Epworth sleepiness scale, mean (SD) | 5.6 (4.1) | 5.7 (4.1) | 5.6 (4.2) | 0.854 |

Data expressed as frequencies and percentages in parenthesis unless otherwise stated. SD: standard deviation; FEV_1_: forced expiratory value in one second; FVC: forced vital capacity.

**Table S2.** *PASHOS Test*.

| ***PASHOS Test*** | |
| --- | --- |
| **Variables** | **Score** |
| **Neck circumference, cm** |  |
| ≤ 35 | 0 |
| > 35 and ≤ 41 | +3 |
| > 41 | +5 |
| **BMI, kg/m^2^** |  |
| ≤ 26 | 0 |
| > 26 and ≤ 30 | +2 |
| > 30 and ≤ 33 | +3 |
| > 33 | +4 |
| **Breathing pauses during sleep** |  |
| No | 0 |
| Yes | +2 |

**Table S3.** Diagnostic accuracy of the *PASHOS Test* as compared with the study questionnaires and with the addition of ODI3%.

| Variables | Sensitivity %  (95% CI) | Specificity %  (95% CI) | Positive predictive value %  (95% CI) | Negative predictive value %  (95% CI) | Overall accuracy %  (95% CI) | Positive likelihood ratio  (95% CI) | Negative likelihood ratio  (95% CI) | Odds ratio  (95% CI) | Post-test probability % (95% CI) |
| --- | --- | --- | --- | --- | --- | --- | --- | --- | --- |
| Questionnaires |  |  |  |  |  |  |  |  |  |
| PASHOS test | 92.6  (83.9-96.8) | 53.2  (44.5-61.7) | 51.6  (42.9-60.3) | 93.1  (84.8-97.0) | 67.0  (60.1-73.2) | 1.98  (1.62-2.41) | 0.14  (0.06-0.33) | 13.31  (5.54-37) | 51.1  (46.2-56.1) |
| Epworth ≥ 11 | 39.7  (28.9-51.6) | 80.8  (73-86.7) | 52.9  (39.5-65.9) | 71.1  (63.2-77.9) | 66.3  (59.4-72.6) | 2.07  (1.3-3.29) | 0.75  (0.6-0.92) | 2.8  (1.4-5.3) | 52.3  (40.8-63.5) |
| Berlin | 72.3  (60.4-81.7) | 52  (43.3-60.7) | 44.3  (35.2-53.8) | 78  (67.9-85.6) | 59  (51.9-65.8) | 1.51  (1.19-1.91) | 0.53  (0.35-0.82) | 2.8  (1.5-5.4) | 44.4  (38.6-50.3) |
| STOP-Bang | 87.7  (77.5-93.6) | 40.2  (31.9-49) | 43.8  (35.6-52.4) | 86  (74.7-92.7) | 56.7  (49.5-63.6) | 1.47  (1.23-1.74) | 0.31  (0.15-0.61) | 4.8  (2.1-10.7) | 43.7  (39.5-47.9) |
| OSA50 | 92.1  (82.7-96.6) | 33.6  (25.7-42.6) | 43  (34.9-51.4) | 88.6  (76-95) | 54.2  (46.9-61.3) | 1.39  (1.2-1.61) | 0.24  (0.1-0.57) | 5.9  (2.2-15.3) | 42.3  (38.8-46) |
| PASHOS test (≥ 5) and ODI3% cut-offs |  |  |  |  |  |  |  |  |  |
| ≥ 10 | 93.5  (84.6-97.4) | 45.8  (33.7-58.3) | 64.4  (54.2-73.6) | 87.1  (71.1-94.9) | 70.2  (61.6-77.7) | 1.72  (1.35-2.2) | 0.14  (0.05-0.38) | 12.2  (4.1-36.3) | 47.7  (41.7-53.8) |
| ≥ 15 | 85.5  (74.7-92.1) | 67.8  (55.1-78.3) | 73.6  (62.4-82.4) | 81.6  (68.6-90) | 76.9  (68.6-83.4) | 2.65  (1.81-3.9) | 0.21  (0.11-0.4) | 12.4  (5.1-29.9) | 58.4  (48.9-67.3) |
| ≥ 20 | 79  (67.4-87.3) | 83.1  (71.5-90.5) | 83.1  (71.5-90.5) | 79  (67.4-87.3) | 81  (73.1-87) | 4.66  (2.61-8.32) | 0.25  (0.15-0.41) | 18.5  (7.5-45.7) | 71.2  (58-81.5) |
| ≥ 25 | 67.7  (55.4-78) | 88.1  (77.5-94.1) | 85.7  (73.3-92.9) | 72.2  (61-81.2) | 77.7  (59.5-84.2) | 5.71  (2.79-11.7) | 0.37  (0.25-0.53) | 15.6  (6.1-39.7) | 75.1  (59.6-86.1) |
| ≥ 30 | 58.1  (45.7-69.5) | 94.9  (86.1-98.3) | 92.3  (79.7-97.3) | 68.3  (57.6-77.4) | 76  (67.7-82.8) | 11.42  (3.72-35.1) | 0.44  (0.33-0.4) | 25.8  (7.7-85.9) | 85.8  (66.3-94.9) |

CI: confidence interval; ODI3%: oxygen desaturation index of 3%.
